# Toxicological Aspects of Methotrexate Intoxication: Concentrations in Postmortem Biological Samples and Autopsy Findings

**DOI:** 10.3390/toxics10100572

**Published:** 2022-09-29

**Authors:** Paweł Szpot, Olga Wachełko, Marcin Zawadzki

**Affiliations:** 1 Department of Forensic Medicine, Wroclaw Medical University, 4 J. Mikulicza-Radeckiego Street, 50345 Wroclaw, Poland; 2 Institute of Toxicology Research, 45 Kasztanowa Street, 55093 Borowa, Poland

**Keywords:** methotrexate, fatal intoxication, poisoning, distribution, LC-MS

## Abstract

The aim of this study was the establishment of a UHPLC-QqQ-MS/MS method to determine methotrexate in postmortem biological samples and quantify the postmortem distribution of methotrexate in a case of fatal intoxication of this drug. A volume of 100 μL or 100 mg of postmortem specimens was precipitated with 400 μL of cold methanol and then analyzed using UHPLC-QqQ-MS/MS. The validation parameters of the method were as follows: limit of quantification: 0.1–1.0 ng/mL or ng/g, coefficient of determination: >0.998 (*R*^2^), matrix effect, intra- and inter-day accuracies and precisions: not greater than 13.6%, 14.8% and 17.4%, respectively. The recoveries were: 89.0–113.6%. The postmortem distribution studies revealed methotrexate concentrations as follows: blood—7.2 ng/mL, vitreous humor—0.8 ng/mL, liver—43.7 ng/g, kidney—20.6 ng/g, bone marrow—29.9 ng/g, lumbar vertebra—20.0 ng/g. The highest concentrations of methotrexate after poisoning were found in the tissues with the most rapidly dividing cells. The method described is simple, precise and selective. Methotrexate concentrations can be routinely determined in postmortem specimens. Determination of methotrexate in the postmortem biological material is possible after a few days of intensive treatment.

## 1. Introduction

Methotrexate (MTX) is a folic acid agonist used as an antineoplastic antirheumatic agent since the 1950s. Methotrexate has many mechanisms of action but the main mechanism is competitive inhibition of the enzyme folic acid reductase. Methotrexate interferes with the growth of certain cells of the body, especially cells that reproduce quickly, such as cancer cells, bone marrow cells, and skin cells. Methotrexate is used to treat leukemia and certain types of cancer of the breast, skin, head and neck, lung, or uterus, and is also used to treat severe psoriasis and rheumatoid arthritis. Methotrexate is used for non-surgical management of ectopic pregnancy and for early pharmacological abortion, because it stops embryonic cells from dividing and multiplying [1]. Methotrexate as a single agent was effective in inducing abortion in early pregnancy [2], but more often it is used in combination with misoprostol [1].

Treatment with methotrexate is often limited due to severe toxicity. Therapeutic drug monitoring (TDM) of methotrexate has been applied in many countries. By examiniation of its pharmacokinetics it is be possible to decrease the risk of toxicity during the therapy. One common pattern of exposure to methotrexate is repeated oral daily dosing [3,4]; a second is accidental acute ingestion [5,6,7]. According to a large series of studies carried out at poisons information centers in Australia [3], France [4], Denmark [8], Spain [9], and the USA [10], the death rate after mistakes in methotrexate dosing range from 5.2% to 24%. It is worth noting that some of the dosing errors are the result of prescription mistakes made by medical personnel [11]. An important here is that improper daily dosing only for at least three days can cause severe complications [3]. A case of pancytopenia and skin damage has been reported after mistakenly taking 5 mg of methotrexate daily for four days [12]. The half-life (t_1/2_) of methotrexate is 5–9 h (low dose) and 16–29 h (high dose) [13]. The bioavailability does not exceed 20% even if a high oral dose is consumed within a few hours [14]. Despite numerous reports of methotrexate poisoning, only one article published to date relates to the forensic science context. That paper described four cases of fatal methotrexate poisoning caused by inappropriate dosing of this drug [15]. However, there was no information about the concentrations of methotrexate in the postmortem material, which is important in terms of forensic toxicology. In the literature the determination of methotrexate in postmortem samples, using liquid chromatography coupled with tandem mass spectrometry, is also lacking. The quantitative determination of methotrexate in biological material is important in the case of poisonings, medical errors and illegal pharmacological abortion. The method presented in this paper was firstly developed for monitoring the blood of fetuses whose mothers were supposed to have used methotrexate for inducing abortion, though we have not detected such a case to date.

To quantify methotrexate in biological samples, liquid chromatography with fluorescence detector [16,17], capillary electrophoresis (CE) with DAD detector [18,19] and liquid chromatography–mass spectrometry (LC–MS/MS) [20,21,22,23,24,25,26,27,28,29,30,31,32,33,34,35] methods have been developed. Methods using CE or HPLC without a mass spectrometry detector show low sensitivity and/or the presence of interfering peaks. Furthermore, sample preparation requires time-consuming extraction (solvent extraction and/or solid-phase extraction).

This paper aims to apply a fast and simple ultra-high-performance liquid chromatography-tandem mass spectrometry with a triple quadrupole (UHPLC-QqQ-MS/MS) method for the determination of methotrexate in postmortem samples. The developed and fully validated method was applied to methotrexate quantification in biological fluids and tissues in a fatal case of intoxication.

## 2. Materials and Methods

### 2.1. Chemicals and Reagents

Water (Chromasolv^®^ LC–MS), acetonitrile (Chromasolv^®^ LC–MS), methanol (Chromasolv^®^ LC–MS) and formic acid were purchased from Sigma-Aldrich (Steinheim, Germany); ammonium formate was purchased from Sigma-Aldrich (Mumbai, India); methotrexate (MTX) hydrate was purchased from Sigma-Aldrich (Gillingham, UK); methotrexate-d_3_ (MTX-d3) was supplied by Cerilliant (Round Rock, TX, USA). Standard solutions of MTX and MTX-d_3_were prepared in methanol. The standard solutions were stored in a refrigerator at −20 °C.

### 2.2. Instrumentation

Chromatographic analysis was performed using an ultra-high performance liquid chromatograph (UHPLC Shimadzu Nexera LC-40 System, Kyoto, Japan). The separation was performed using a Kinetex XB-C18 column (100 × 2.1 mm i.d., particle size 2.6 μm; Phenomenex, Torrance, CA, USA) with a guard column SecurityGuard Ultra C18 (15 × 2.1 mm; Phenomenex) with a thermostat at 40 °C. A mixture of 10 mM ammonium formate/0.1% formic acid in water (A) and 10 mM ammonium formate/0.1% formic acid in methanol (B) was used as a mobile phase. The gradient elution was carried out at constant flow 0.3 mL/min. The gradient applied was the following: 0 min, 5% B; 7.5 min, 95% B; and 10 min, 95% B. Return to the initial gradient composition (95% A and 5% B) was performed for 5 min. The injected volume was 2 μL.

Detection of the methotrexate was achieved using a triple-quadrupole mass spectrometer (QqQ, Shimadzu 8060, Kyoto, Japan). The spectrometer was equipped with an ESI source; determination of the methotrexate was carried out in the MRM mode. The following MS parameters were fixed: nebulizing gas flow: 3 L/min, heating gas slow: 10 L/min, interface temperature: 300 °C, DL temperature: 250 °C, heat block temperature: 400 °C, drying gas flow: 10 L/min. A summary of precursor and product ions, collision energies, dwell time, Q1–Q3 pre bias voltages and retention time for each compound is presented in Table 1.

### 2.3. Blank Samples

Blank samples of postmortem human blood, vitreous humor, kidney and liver were collected during autopsies performed in the Department of Forensic Medicine. Blank samples did not contain any anticoagulant and were screened prior to spiking to ensure that they were free from methotrexate. Postmortem biological samples collected from the authentic fatal intoxication case (blood, vitreous humor, liver, kidney, bone marrow and lumbar vertebra) were sent to our laboratory for routine toxicological analysis.

### 2.4. Sample Preparation

We transferred 100 μL of liquid samples (postmortem blood, vitreous humor or bone marrow) to a 2-mL Eppendorf tube. Next, 10 μL of internal standard solution (methotrexate-*d_3_* at concentration of 1000 ng/mL) was added. Samples were mixed with 400 μL of cold MeOH (stored on ice) and vortex-mixed to precipitate the proteins. The precipitated proteins were removed by centrifugation at 13,500× *g* rpm for 10 min (at 4 °C). Aliquot (100 μL) of the clear supernatant was transferred into glass inserts of the autosampler vials and analyzed by UHPLC-QqQ-MS/MS.

Solid tissue (1 g) was transferred to 12-mL plastic tube. Next, 1 mL of water (Chromasolv^®^ LC–MS grade) was added and the sample was homogenized using a Q55 sonicator (QSonica, Newtown, CT, USA). Next, 100 μL of the homogenate was subjected to the same procedure as the liquid samples.

### 2.5. Working Solutions, Calibration Curve, Quality Control Samples

Standard solutions were diluted with methanol to obtain working standard solutions at the following concentrations of methotrexate: 1, 2, 2.5, 5, 10, 50, 100, 500, 1000, 5000 and 10,000 ng/mL. Quality control (QC) samples and calibration points were prepared by diluting the appropriate working solution with human samples (blood, vitreous humor as well as homogenates of kidney and liver). The final concentrations of the calibrators were: 0.1, 0.2, 0.25, 0.5, 1, 5, 10, 50, 100, 500 and 1000 ng/mL (biological fluids) or ng/g (solid tissues) of methotrexate in samples. QC samples were prepared by spiking blank human liquid or solid tissues to yield final concentrations of 1 (low QC), 100 (medium QC) and 1000 (high QC) ng/mL or ng/g for methotrexate.

### 2.6. Method Validation

Evaluated parameters of the method included examination of selectivity, linearity, precision and accuracy, lower limit of quantification, recovery and matrix effect. The validation of the method was performed in accordance with GTFCh (Gesellschaft für Toxikologische und Forensische Chemie ang. *German Society of Toxicological and Forensic Chemistry*) recommendations.

#### 2.6.1. Selectivity

Five different lots of each blank biological matrix (blood, vitreous humor, kidney, liver) were tested for possible endogenous interference peaks at the retention time of the methotrexate.

#### 2.6.2. Linearity

Linearity was evaluated by the analysis of methotrexate working solution with human postmortem samples in final concentrations of 0.1, 0.2, 0.25, 0.5, 1, 5, 10, 50, 100, 500, and 1000 ng/mL or ng/g. Linearity of the calibration curve was determined by plotting the peak area ratio of methotrexate to IS in human postmortem samples with concentration for assessment of method performance. The calibration range was determined as the range where all calibration levels were within 80–120% of the theoretical concentration. The coefficients of determination (*R*^2^) were determined for each tested biological matrix.

#### 2.6.3. Precision and Accuracy

The precision and accuracy of the method were estimated by replicating the analysis (*n* = 5) of QC samples at three concentration levels: 1 (low QC), 100 (medium QC) and 1000 (high QC) ng/mL or ng/g. Intraday precision and accuracy were evaluated by analyzing QC samples five times over one day, while inter-day precision and accuracy were estimated by analyzing QC samples once in each of the five subsequent days. The precision and accuracy were defined as relative standard deviation (RSD%) and relative error (RE%), respectively.

#### 2.6.4. Lower Limits of Quantification (LLOQ)

LLOQ was defined as the minimal concentration at which the relative standard deviation (RSD%) does not exceed 20%.

#### 2.6.5. Recovery and Matrix Effect

The recovery of the methotrexate was evaluated at each of the three different concentrations 1, 100 and 1000 ng/mL or ng/g. The recovery (%, *n* = 5) was determined by comparing the response of the extracted analyte in a spiked blank matrix with the response of the analyte spiked after the extraction of the blank matrix. The matrix effect (%bias, *n* = 5) was determined by comparing the response of the analyte spiked after the extraction of the blank matrix with the response of the analyte in a neat solution [36].

### 2.7. Case Histories

#### 2.7.1. Case 1

**Figure 1:**
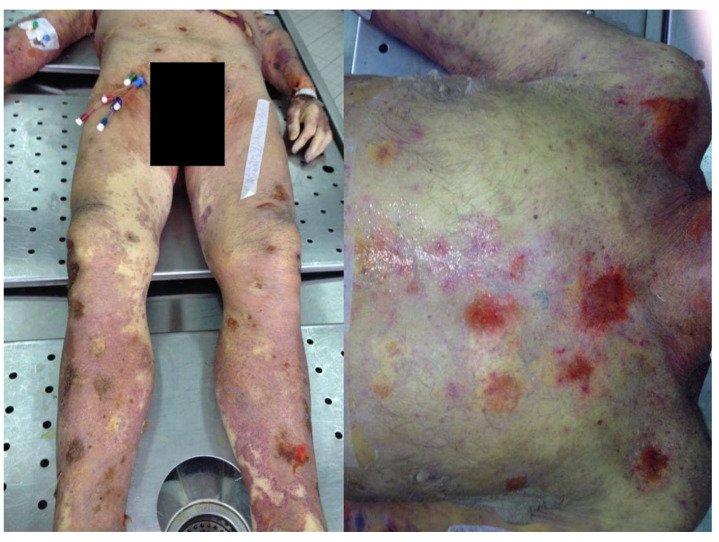
Multiple petechial hemorrhages on the body surface.

A 71-year-old male was admitted to the hospital presenting rapidly developing pancytopenia. His state was primarily assessed as critical. The elicited history revealed erroneous dosing of methotrexate, with the prescribed weekly dose being administered daily. A thorough skin examination exposed extensive psoriatic lesions, widespread erythema and multiple epithelial exfoliation. An immediate resuscitation with pressor amines and instrumented airway management had been commenced at admission and due to anuric acute kidney injury an emergency renal replacement therapy (RRT) had been implemented immediately. Both biochemical blood analysis and complete blood count showed substantial abnormalities, predominantly leukopenia and trombocytopenia. Based upon both clinical symptoms and laboratory values, the diagnosis of septic shock was made, a blood cultures set was collected and subsequently empiric antimicrobial therapy was administered. Even so, the patient was pronounced dead a few hours after admission. The postmortem examination revealed toxic epidermal necrolysis affecting both skin and mucosae with extensive petechial hemorrhages (Figure 1), marked jaundice, psoriatic lesions, thyroid goitre, left ventricular hypertrophy (LVH), advanced atherosclerosis, cortical renal cysts, benign prostatic hyperplasia (BPH) and nodular adrenal hyperplasia. Key findings in the histopathological study comprised moderate intracellular cholestasis and acute tubular injury (ATI) following tubulointerstitial nephritis.

#### 2.7.2. Case 2

A 76-year-old female was admitted to the hospital for the management of iatrogenic pancytopenia after accidental methotrexate overdose, which resulted in bilateral pneumonia. Despite the prescribed dosage being 15 mg per week, the accumulated dose was 55 mg (2.5 mg tablet twice a day for 11 days of treatment). The protective folic acid prophylaxis was not used. Five days prior to admission, the patient presented with a 39 °C fever, sore throat, labial oedema, excessive salivation with blood admixture. All of the above was accompanied by recto-vaginal pains. The ER doctor assessed the patient’s condition as severe, as more hemorrhagic–necrotic lesions were revealed in the oral cavity, within the perianal skin, lower extremities and back. Laboratory tests revealed severe pancytopenia in conjunction with electrolyte imbalance, hypoproteinemia and coagulopathy. Aplastic histology of the bone marrow supported the diagnosis. The clinicians were able to achieve partial bone marrow reconstitution using granulocyte-macrophage colony-stimulating factor (GM-CSF), although repeated red blood cell and platelet concentrate transfusions, as well as albumin infusions and fresh frozen plasma administration were crucial. Despite the intensive care, the patient suffered from combined *Enterococcus faecium*, *Stenotrophomonas maltophilia* and *Aspergillus fumigatus* sepsis and was not susceptible to further resuscitation. The following 10 days brought an exacerbation of respiratory failure, followed by mechanical ventilation commencement, which was further complicated by right-sided hemopneumothorax. Rapid drainage failed to prevent increasing bradycardia, persistent hypotension and eventual sudden cardiac arrest.

#### 2.7.3. Case 3

This involves suspected methotrexate poisoning of a woman who was hospitalized with symptoms of extensive skin and bone marrow damage. The patient died due to multiple organ failure after several days of intensive therapy with blood transfusions. Unfortunately, the authors in this case did not have the dossier, including the medical history.

**Figure 2:**
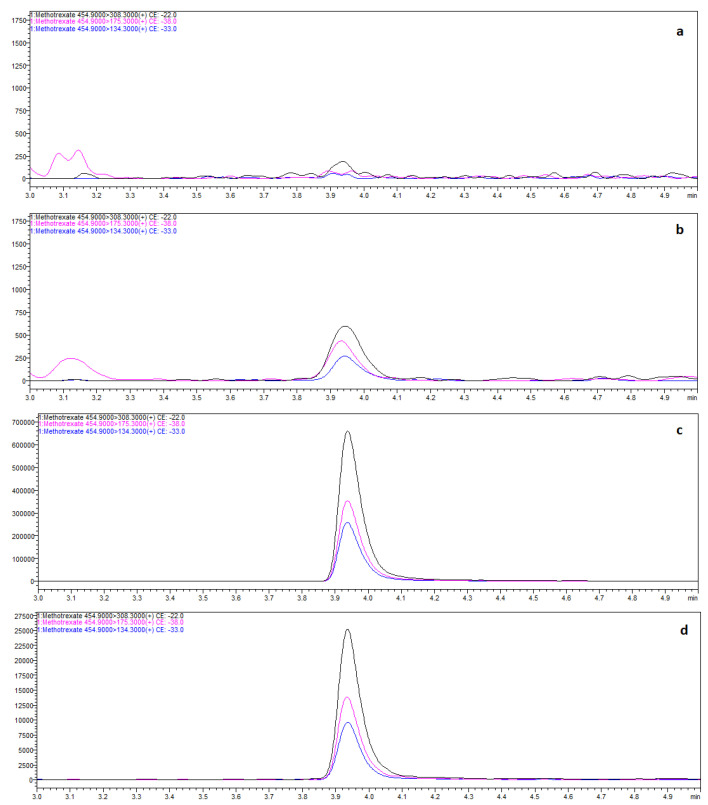
MRM of methotrexate in blank human postmortem blood (**a**), 0.1 ng/mL—low QC (**b**), 100 ng/mL—medium QC (**c**) and real sample of human blood with methotrexate at concentration of 7.2 ng/mL—case 3 (**d**).

An autopsy was performed, and postmortem biological fluids (blood, bone marrow and vitreous humor) and tissues (liver, kidney and lumbar vertebra) were collected. All samples were measured in duplicate and mean values were calculated. The chromatogram of blood sample from this case is shown in Figure 2d. Determination of the methotrexate in bone marrow and lumbar vertebra was performed on the blood and liver calibration curves, respectively.

## 3. Results and Discussion

### 3.1. Method Development

**Figure 3:**
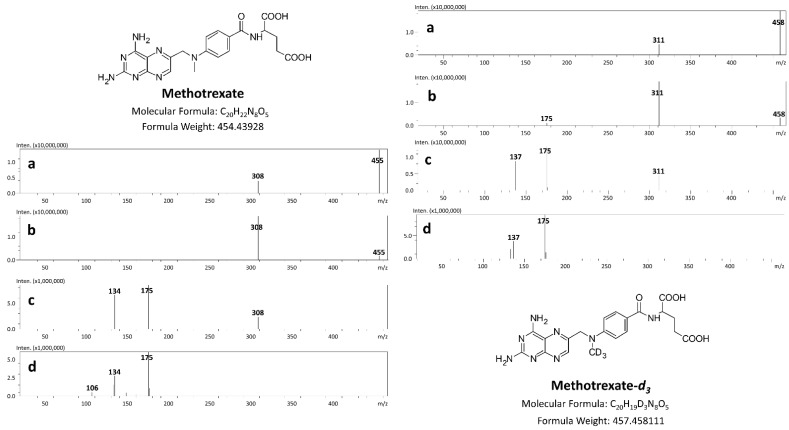
Product ion scan spectra of methotrexate and methotrexate-*d*_3_ at different collision energies: (**a**) 10 V; (**b**) 20 V; (**c**) 35 V and (**d**) 50 V.

The product ion scan spectra of methotrexate and methotrexate-*d_3_* are presented in Figure 3. Under the chromatographic conditions the *m/z* transitions of 454.9 → 308.30, 454.9 → 175.30, 454.9 → 134.30 and 457.90 → 311.35, 457.90 → 175.25, 457.90 → 137.35 were selected for optimal monitoring of methotrexate and methotrexate-*d*_3_, respectively. A simple precipitation with methanol was successfully applied to extract the methotrexate and IS from postmortem materials.

The linear concentration range was from 0.1 to 1000 ng/mL or ng/g for methotrexate in the blood and kidney. The coefficient of determination values (*R*^2^) were >0.996 for all matrix. The recovery, matrix effects, intra-day and inter-day precision and accuracy values are presented in Table 2. The intra-day RSD% data obtained from five repetitive measurements of blood, vitreous humor, kidney and liver at three concentration levels (1, 100, 1000 ng/mL or ng/g of methotrexate) ranged from 0.5% to 17.4%. The inter-day RSD% ranged from 0.3% to 14.3%. The results for intra- and inter-day accuracy at the three quality control levels were found to be less than 14.8%. Based on the above results, we conclude that our method is sufficiently accurate and precise to be used in routine forensic toxicological analysis. The mean recovery values were in a range from 89.0% to 113.6%. Regarding the matrix effects, all concentrations ranged from −11.0% to 13.6% of the nominal values, suggesting that there were no significant matrix effects for methotrexate.

The comparison of LC-MS methods for determination of methotrexate in biological samples is shown in Table 3. In all studies, liquid chromatography coupled with a triple quadrupole spectrometer or a Q-trap spectrometer was used. One article described the determination of methotrexate in saliva [24], one in cerebrospinal fluid [27], one in serum [31], one in whole blood [32], three in urine [20,33,34] and eleven in plasma [20,21,22,23,25,26,27,28,29,30,35]. The fact that most methods compared in Table 3 utilize plasma as the biological matrix is most understandable because these methods have been developed for controlling the methotrexate concentrations in clinical toxicology practice. In nine articles [20,22,27,28,29,31,33,34,35] an isotope-labeled standard of methotrexate was used as an internal standard, meaning the analysis resulted in a high recovery of methotrexate in most methods. However, the method described by Bouqui’e et al. [28] was characterized by a very poor recovery value (24%) despite the fact that methotrexate-*d_3_* was used as an IS. The most popular extraction method was SPE [20,23,24,27,30,34,35], followed by protein precipitation [21,25,28,29,31,33], and LLE [22,26]. It is worth mentioning that one method combined two preanalytical techniques—protein precipitation and LLE [32]. The SPE and LLE methods usually involve complex sample pretreatment with sample extraction and evaporation of the organic phase for enrichment purposes. The most sensitive of the presented methods is that proposed by Canal-Raffin et al. [34]; however, the authors used SPE (which is time-consuming), tested urine (a less complex matrix than postmortem samples) and utilized a fifty-fold greater biological material volume in comparison with the procedure described in this paper. It is difficult to compare methods using different matrices and different sample volumes. Only one of the articles included in Table 3 presented a method for the determination of methotrexate in whole blood [32]. Other methods were developed for testing other biological matrices. In forensic toxicology, we rarely have plasma or serum for testing, so it is important to have a precise, sensitive and accurate method for quantification of methotrexate in the postmortem blood. Mo et al. [32] have developed a very interesting method for quantifying methotrexate in blood, but it requires quite large sample volume (four times more biological material than the procedure applied in our method). In addition, the method described by Mo et al. is not as sensitive as the one described in this paper, while the sample preparation based on two-step sample pretreatment (precipitation followed by LLE) makes it more difficult to conduct and more time-consuming. The development of a sensitive method for the determination of methotrexate in postmortem blood is important especially as we expect that some patients may die of sepsis, pneumonia or multi-organ failure several days (5–14 days) after intoxication [11]. In addition, a small amount of postmortem blood is extremely important in cases of toxicological examination of blood samples from newborns or miscarried fetuses. In such cases, it is difficult to collect a large amount of biological material.

### 3.2. Case Analysis

Methotrexate (MTX) has been a well-known and efficacious conventional disease-modifying antirheumatic drug (DMARD) in rheumatoid arthritis for several decades. In accordance with current medical knowledge, adverse drug reactions can occur even after ingestion of low doses. Similarly to what other forensic practitioners have reported in their paper [15], two of the abovementioned intoxication cases focused on incorrect drug dosage. (However, despite the unavailability of medical information in case 3, such a cause of death could not be excluded.) Common methotrexate intoxication symptoms include severe myelosuppression, ulceration of the mucous membranes, nausea, vomiting, stomatitis and acute liver failure. As far as acute kidney injury (AKI) is concerned, methotrexate promotes intrarenal crystal formation. This process results in the development of an intrinsic type of AKI. This, in turn, impairs the renal clearance of the xenobiotic and consequently causes an increase in its serum levels [14]. AKI was diagnosed only in *Case 1*. All patients presented pancytopenia and petechial hemorrhages, both of which cause damage to rapidly dividing cells.

Samples from Patients 1 and 2 were inaccessible for the authors, since biological material for toxicology examinations was collected solely in *Case 3*. It is worth noticing the fact that, despite fluid resuscitation and numerous transfusions, methotrexate poisoning was confirmed by UHPLC-QqQ-MS/MS analysis. Concentrations of methotrexate in postmortem biological samples collected during the autopsy are presented in Table 4. *Case 3* proves that MTX can be determined in postmortem biological samples even a few days after the ingestion of the last dose, which may be of key importance if an expert opinion on the confirmation of poisoning is to be obtained.

As shown in Table 4, the concentrations in the solid tissue specimens were one order of magnitude higher than those obtained from the blood and vitreous humor specimens. The highest MTX concentrations were found in the liver, kidney and bone marrow samples. The conducted studies prove that bone marrow samples, which are relatively easy to access, are a valuable biological material for the detection of methotrexate and a wide range of xenobiotics in general, as mentioned earlier [37].

The MTX concentrations assessed in our study are manifestly below those perceived as therapeutic, but they still prove that the highest levels of the drug are found in most rapidly dividing tissues. We confirm this also on the grounds of the low levels of the xenobiotic in hypocellular tissue, such as vitreous humor.

Research from the 1970s on methotrexate tissue distribution also indicated that the highest concentrations are noted in liver, kidney, gall bladder, spleen and skin appendages [38]. Conversely, one patient (*Case 3*) received a great volume of balanced crystalloids and blood component transfusions, which could possibly affect blood concentrations.

In view of methotrexate’s high toxic potential, it should be considered extremely important to increase public awareness of its toxicity among healthcare practitioners, pharmacists and patients receiving such treatment.

## 4. Conclusions

The presented method is fast, simple and sensitive due to the use of a mass spectrometer in MRM mode. To quantify the postmortem distribution of methotrexate, we established a detailed procedure for UHPLC-QqQ-MS/MS analysis with a simple and fast sample preparation method. Determination of methotrexate in the postmortem biological material is possible even after a few days of intensive medical treatment and blood transfusions. The highest concentrations of methotrexate after fatal poisoning were found in the tissues with the most rapidly dividing cells. Considering the fast preanalytical procedure of methotrexate’s determination (the sample preparation involves only precipitation of proteins with cold methanol and centrifugation), as well as the possibility of applying this technique to other types of biological materials such as plasma or serum (which are routinely obtained in clinical toxicology), we believe that the established UHPLC-QqQ-MS/MS method can be successfully applied in terms of monitored therapy with methotrexate.

## Figures and Tables

**Table 1:** UHPLC–ESI-QqQ-MS/MS parameters for MTX and MTX-*d*_3_.

Compounds	Retention Time ^a^	Precursor Ions ^b^	Product Ions ^b^	Dwell Time ^c^	Q1 Pre Bias ^d^	CE ^d^	Q3 Pre Bias ^d^
MTX	3.94	454.9	308.3 * 175.3 134.3	22	−13 −13 −13	−22 −38 −33	−21 −11 −24
MTX-*d_3_*	3.96	457.9	311.3 * 175.2 137.3	22	−13 −13 −13	−21 −45 −33	−22 −11 −19

^a^ minutes (min.); ^b^ mass to charge ratio (*m*/*z*); ^c^ millisecond (msec); ^d^ voltage (V); * Ions selected for quantitative analysis.

**Table 2:** Calibration curves parameters, LLOQ, recoveries, matrix effects, intra- and inter-day precision and accuracy of the UHPLC-QqQ-MS/MS method for determination of methotrexate in postmortem fluids and tissues.

Biological Matrix	Validation Parameters									
	The Linear Concentration Range [ng/mL] or [ng/g]	The Coefficient of Determination (*R*^2^)	LLOQ [ng/mL] or [ng/g]	Concentration Level [ng/mL] or [ng/g]	Intraday		Interday		Recovery [%] *	Matrix Effect [%] *
					Precision [%] *	Accuracy [%] *	Precision [%] *	Accuracy [%] *		
Postmortem blood	0.1–1000	>0.998	0.1	1 100 1000	7.7 5.5 4.5	14.3 5.5 5.1	10.8 14.0 10.3	4.9 11.6 8.6	104.7 101.8 89.0	4.7 1.8 −11.0
Vitreous humor	0.5–1000	>0.996	0.5	1 100 1000	2.0 7.2 17.4	9.1 11.5 11.0	13.4 8.4 5.7	9.3 5.1 14.8	113.6 105.0 101.2	13.6 1.2 1.2
Kidney	0.1–1000	>0.999	0.1	1 100 1000	5.2 6.4 4.6	7.8 0.3 3.8	0.8 4.3 2.4	−0.4 −0.9 −0.5	106.7 97.7 100.3	6.7 −2.3 0.3
Liver	1.0–1000	>0.999	1.0	1 100 1000	0.5 1.5 2.6	0.5 1.5 8.3	6.4 4.0 12.7	7.0 4.1 5.9	99.4 92.2 100.4	−0.6 −7.8 0.4

* *n* = 5.

**Table 3:** Comparison of LC-MS methods for determination of methotrexate in biological samples.

Type of Biological Sample (Volume)	Sample Preparation Technique	Detector (Mode)	Recovery (%)/IS	LOQ/Unit	References
Plasma (500 μL)	SPE	ESI/triple quadrupole (MRM)	94.28–102.22/thiouracil	6.25 ng/mL	[23]
Saliva (1400 μL)	SPE	ESI/Qtrap (MRM)	89–94/ aminopterin	2 ng/mL ^a^	[24]
Plasma and urine (30 μL)	SPE	Z-Spray/triple quadrupole (MRM)	≥95/ methotrexate-*d*_3_	1000 ng/mL	[20]
Plasma (10 μL)	Protein precipitation with methanol	ESI/triple quadrupole (MRM)	66.2/ aminopterin	3.7 ng/mL	[21]
Plasma (200 μL)	LLE with chloroform	ESI/triple quadrupole (MRM)	61.0/ methotrexate-*d*_3_	0.5 ng/mL	[22]
Plasma (10 μL)	Dilution with water– acetonitrile, 70:30 (*v*/*v*)	ESI/Qtrap (MRM)	97.8–112.0/ *p*-amino-acetophenone	0.05 μmol/L	[25]
Plasma (50 μL)	LLE with *tert*-methyl-butyl ether	HESI/triple quadrupole (MRM)	79.4–87.2/tolbutamide	1 ng/mL	[26]
Plasma and cerebrospinal fluid (100 μL)	SPE	ESI/Qtrap (MRM)	86.5–90.4 (plasma) 98.7–103.8 (CSF)/methotrexate-*d*_3_	0.0022 μM	[27]
Plasma (50 μL)	Protein precipitation with methanol and 0.2 M ZnSO_4_ (80:20, *v*/*v*)	ESI/triple quadrupole (MRM)	24.0/ ^13^C_2_H_3_-methotrexate	0.025 μmol/L	[28]
Plasma (50 μL)	Protein precipitation with cold 16% perchloric acid	ESI/triple quadrupole (MRM)	96.0–102.0/methotrexate-*d*_3_	5 nM	[29]
Plasma (50 μL)	SPE	ESI/Qtrap (MRM)	84.8–90.7/phenacetin	0.49 ng/mL	[30]
Plasma (200 μL)	SPE	ESI/triple quadrupole (MRM)	92.0/ methotrexate-*d*_3_	0.5 ng/mL	[35]
Serum (100 μL)	Protein precipitation with methanol containing formic acid	HESI/triple quadrupole (MRM)	94.6–104.3/methotrexate-*d*_3_	10 nmol/L	[31]
Whole blood (400 μL)	Protein precipitation with trifluoroacetic acid solution and LLE with ethyl acetoacetate	ESI/triple quadrupole (MRM)	29.3–37.8/doxofylline	1 ng/mL	[32]
Urine (50 μL)	Protein precipitation with ACN	ESI/triple quadrupole (MRM)	104.0–126.0/methotrexate-*d*_3_	2.5 nM	[33]
Urine (5000 μL)	SPE	ESI/triple quadrupole (MRM)	75.3–81.7/methotrexate-*d*_3_	20 pg/mL	[34]
Postmortem samples (100 μL or 100 mg)	Protein precipitation with methanol	ESI/triple quadrupole (MRM)	89.0–113.6/methotrexate-*d*_3_	0.1–1.0 ng/mL or ng/g	Presented method

^a^ Expressed as LCL (lower calibration level). **Abbreviations:** ESI—electrospray ionization; HESI—heated-electrospray ionization; MRM—multiple reaction monitoring; ACN—acetonitrile; SPE—solid-phase extraction; LLE—liquid-liquid extraction; LOQ—limit of quantification; Qtrap—quadrupole-linear ion trap.

**Table 4:** Concentrations of MTX in body fluids and solid tissues of the deceased.

	Blood	Vitreous Humor	Liver	Kidney	Bone Marrow	Lumbar Vertebra
MTX concentrations [ng/mL] or [ng/g] *	7.2	0.8	43.7 *	20.6 *	29.9 *	20.0 *

Concentrations provided in ng/g were marked by asterisk (*).

## References

[1] Borgatta L., Burnhill M.S., Tyson J., Leonhardt K.K., Hausknecht R.U., Haskell S. (2001). Early medical abortion with methotrexate and misoprostol. Obstet. Gynecol..

[2] Schaff E.A., Penmetsa U., Eisinger S.H., Franks P. (1997). Methotrexate. A single agent for early abortion. J. Reprod. Med..

[3] Cairns R., Brown J.A., Lynch A.M., Robinson J., Wylie C., Buckley N.A. (2016). A decade of Australian methotrexate dosing errors. Med. J. Aust..

[4] Vial T., Patat A.M., Boels D., Castellan D., Villa A., Theophile H., Torrents R., Kassai B. (2019). Adverse consequences of low-dose methotrexate medication errors: Data from French poison control and pharmacovigilance centers. Jt. Bone Spine.

[5] Hays H., Beuhler M.C., Spiller H.A., Weber J., Mowry J.B., Ryan M.L., Spiller N.E., Webb A. (2018). Evaluation of toxicity after acute accidental methotrexate ingestions in children under 6 years old: A 16-year multi-center review. Clin. Toxicol..

[6] Badurdeen S., Kang S.L., Saravanan M. (2011). Accidental methotrexate ingestion in a 19-month-old child. BMJ Case Rep..

[7] Gibbon B.N., Manthey D.E. (1999). Pediatric case of accidental oral overdose of methotrexate. Ann. Emerg. Med..

[8] Perregaard H., Aronson J.K., Dalhoff K., Hellebek A. (2015). Medication errors detected in non-traditional databases: Types of errors in methotrexate dosing as listed in four different Danish registers. Eur. J. Clin. Pharmacol..

[9] Salgueiro-Vázquez M.E., Sáinz Gil M., Fernández Peña S., Martín Arias L.H. (2017). Medication errors associated with oral administration of methotrexate. Data from spontaneous reporting and medical literature review. Med. Clin..

[10] Moore T.J., Walsh C.S., Cohen M.R. (2004). Reported medication errors associated with methotrexate. Am. J. Health Syst. Pharm..

[11] Sinicina I., Mayr B., Mall G., Keil W. (2005). Deaths following methotrexate overdoses by medical staff. J. Rheumatol..

[12] Singh A., Handa A.C. (2018). Medication Error—A Case Report of Misadventure with Methotrexate. JNMA.

[13] Baselt R.C. (2017). Disposition of Toxic Drugs and Chemical in Man.

[14] Chan B.S., Dawson A.H., Buckley N.A. (2017). What can clinicians learn from therapeutic studies about the treatment of acute oral methotrexate poisoning?. Clin. Toxicol..

[15] Moisa A., Fritz P., Benz D., Wehner H.D. (2006). Iatrogenically-related, fatal methotrexate intoxication: A series of four cases. Forensic Sci. Int..

[16] Uchiyama M., Matsumoto T., Matsumoto T., Jimi S., Takamatsu Y., Tamura K., Hara S. (2012). Simple and sensitive HPLC method for the fluorometric determination of methotrexate and its major metabolites in human plasma by post-column photochemical reaction. Biomed. Chromatogr..

[17] Li H., Luo W., Zeng Q., Lin Z., Luo H., Zhang Y. (2007). Method for the determination of blood methotrexate by high performance liquid chromatography with online post-column electrochemical oxidation and fluorescence detection. J. Chromatogr. B Anal. Technol. Biomed Life Sci..

[18] Rodríguez Flores J., Peñalvo G.C., Mansilla A.E., Gómez M.J. (2005). Capillary electrophoretic determination of methotrexate, leucovorin and folic acid in human urine. J. Chromatogr. B Anal. Technol. Biomed. Life Sci..

[19] Kuo C.Y., Wu H.L., Kou H.S., Chiou S.S., Wu D.C., Wu S. (2003). Simultaneous determination of methotrexate and its eight metabolites in human whole blood by capillary zone electrophoresis. J. Chromatogr. A.

[20] Rule G., Chapple M., Henion J. (2001). A 384-well solid-phase extraction for LC/MS/MS determination of methotrexate and its 7-hydroxy metabolite in human urine and plasma. Anal. Chem..

[21] Guo P., Wang X., Liu L., Belinsky M.G., Kruh G.D., Gallo J.M. (2007). Determination of methotrexate and its major metabolite 7-hydroxymethotrexate in mouse plasma and brain tissue by liquid chromatography-tandem mass spectrometry. J. Pharm. Biomed. Anal..

[22] Steinborner S., Henion J. (1999). Liquid-liquid extraction in the 96-well plate format with SRM LC/MS quantitative determination of methotrexate and its major metabolite in human plasma. Anal. Chem..

[23] Al-Ghobashy M.A., Hassan S.A., Abdelaziz D.H., Elhosseiny N.M., Sabry N.A., Attia A.S., El-Sayed M.H. (2016). Development and validation of LC-MS/MS assay for the simultaneous determination of methotrexate, 6-mercaptopurine and its active metabolite 6-thioguanine in plasma of children with acute lymphoblastic leukemia: Correlation with genetic polymorphism. J. Chromatogr. B Anal. Technol. Biomed. Life Sci..

[24] Rodin I., Braun A., Stavrianidi A., Shpigun O. (2013). A validated LC-MS/MS method for rapid determination of methotrexate in human saliva and its application to an excretion evaluation study. J. Chromatogr. B Anal. Technol. Biomed. Life Sci..

[25] Wu D., Wang Y., Sun Y., Ouyang N., Qian J. (2015). A simple, rapid and reliable liquid chromatography-mass spectrometry method for determination of methotrexate in human plasma and its application to therapeutic drug monitoring. Biomed. Chromatogr..

[26] Thappali S.R., Varanasi K.V., Veeraraghavan S., Vakkalanka S.K., Khagga M. (2012). Simultaneous determination of methotrexate, dasatinib and its active metabolite N-deshydroxyethyl dasatinib in rat plasma by LC-MS/MS: Method validation and application to pharmacokinetic study. Arzneimittelforschung.

[27] Roberts M.S., Selvo N.S., Roberts J.K., Daryani V.M., Owens T.S., Harstead K.E., Gajjar A., Stewart C.F. (2016). Determination of Methotrexate, 7-Hydroxymethotrexate, and 2,4-Diamino-N10-methylpteroic Acid by LC-MS/MS in Plasma and Cerebrospinal Fluid and Application in a Pharmacokinetic Analysis of High-Dose Methotrexate. J. Liq. Chromatogr. Relat. Technol..

[28] Bouquié R., Deslandes G., Bernáldez B.N., Renaud C., Daillyad E., Jolliet P. (2014). A fast LC-MS/MS assay for methotrexate monitoring in plasma: Validation, comparison to FPIA and application in the setting of carboxypeptidase therapy. Anal. Methods.

[29] den Boer E., Heil S.G., van Zelst B.D., Meesters R.J., Koch B.C., Te Winkel M.L., van den Heuvel-Eibrink M.M., Luider T.M., de Jonge R. (2012). A U-HPLC-ESI-MS/MS-based stable isotope dilution method for the detection and quantitation of methotrexate in plasma. Ther. Drug Monit..

[30] Sharma K., Giri K., Dhiman V., Dixit A., Zainuddin M., Mullangi R. (2015). A validated LC-MS/MS assay for simultaneous quantification of methotrexate and tofacitinib in rat plasma: Application to a pharmacokinetic study. Biomed. Chromatogr..

[31] Schofield R.C., Ramanathan L.V., Murata K., Grace M., Fleisher M., Pessin M.S., Carlow D.C. (2015). Development and validation of a turbulent flow chromatography and tandem mass spectrometry method for the quantitation of methotrexate and its metabolites 7-hydroxy methotrexate and DAMPA in serum. J. Chromatogr. B Anal. Technol. Biomed. Life Sci..

[32] Mo X., Wen Y., Ren B., Chen J., Xu C., Wang X., Huang M., Chen X. (2012). Determination of erythrocyte methotrexate polyglutamates by liquid chromatography/tandem mass spectrometry after low-dose methotrexate therapy in Chinese patients with rheumatoid arthritis. J. Chromatogr. B Anal. Technol. Biomed. Life Sci..

[33] Bluett J., Riba-Garcia I., Hollywood K., Verstappen S.M., Barton A., Unwin R.D. (2015). A HPLC-SRM-MS based method for the detection and quantification of methotrexate in urine at doses used in clinical practice for patients with rheumatological disease: A potential measure of adherence. Analyst.

[34] Canal-Raffin M., Khennoufa K., Martinez B., Goujon Y., Folch C., Ducint D., Titier K., Brochard P., Verdun-Esquer C., Molimard M. (2016). Highly sensitive LC-MS/MS methods for urinary biological monitoring of occupational exposure to cyclophosphamide, ifosfamide, and methotrexate antineoplastic drugs and routine application. J. Chromatogr. B Anal. Technol. Biomed Life Sci..

[35] Upadhyay V., Rajput M., Sen A., Suvarna S., Dhanse S. (2017). A sensitive, high throughput estimation of methotrexate in human plasma by high performance liquid chromatography tandem mass spectrometry. Int. J. Pharm. Sci. Res..

[36] Chambers E., Wagrowski-Diehl D.M., Lu Z., Mazzeo J.R. (2007). Systematic and comprehensive strategy for reducing matrix effects in LC/MS/MS analyses. J. Chromatogr. B Analyt. Technol. Biomed Life Sci..

[37] Iskierka M., Zawadzki M., Szpot P., Jurek T. (2021). Detection of Drugs in Postmortem Specimens of Blood, Vitreous Humor and Bone Marrow Aspirate. J. Anal. Toxicol..

[38] Anderson L.L., Collins G.J., Ojima Y., Sullivan R.D. (1970). A study of the distribution of methotrexate in human tissues and tumors. Cancer Res..

